# Integrative analysis of DNA methylation, RNA sequencing, and genomic variants in the cancer genome atlas (TCGA) to predict endometrial cancer recurrence

**DOI:** 10.3389/fgene.2025.1569122

**Published:** 2025-04-28

**Authors:** Jin Hwa Hong, Yung Taek Ouh, Sohyeon Jeong, Yoonji Oh, Hyun Woong Cho, Jae Kwan Lee, Hayeon Kim, Chungyeul Kim, Sanghyun Roh, Eun Na Kim, Yikyeong Chun, Jeong-An Gim

**Affiliations:** ^1^ Department of Obstetrics and Gynecology, Guro Hospital, Korea University College of Medicine, Seoul, Republic of Korea; ^2^ Department of Obstetrics and Gynecology, Ansan Hospital, Korea University College of Medicine, Ansan, Republic of Korea; ^3^ Department of Pathology, Guro Hospital, Korea University College of Medicine, Seoul, Republic of Korea; ^4^ Department of Medical Science, Soonchunhyang University, Asan, Republic of Korea; ^5^ Departments of Pathology, Seoul National University Hospital, Seoul National University College of Medicine, Seoul, Republic of Korea

**Keywords:** endometrial cancer, machine-learning, recurrence, the cancer genome atlas, multiomics analysis

## Abstract

**Introduction:**

The prognosis within each subtype varies due to histological and molecular factors. This study leverages omics datasets and machine learning to identify biomarkers associated with EC recurrence in different molecular subtypes.

**Methods:**

Utilizing DNA methylation, RNA-sequencing, and common variant data from 116 EC samples in The Cancer Genome Atlas (TCGA), differentially expressed genes (DEGs) and differentially methylated regions (DMRs) were identified using t-tests between recurrence and non-recurrence groups. These were visualized through volcano plots and heat maps, while decision trees and random forests classified and stratified the samples.

**Results:**

A machine learning analysis combined with box plots showed that in the copy number-high (CN-H) recurrence group, PARD6G-AS1 had decreased methylation, CSMD1 had increased methylation, and TESC expression was higher than the non-recurrence group. In the copy number-low (CN-L) recurrence group, CD44 expression was elevated. Further validation using TCGA clinical data confirmed PARD6G-AS1 hypomethylation and CD44 overexpression as significant indicators of recurrence (p=0.006 and p=0.02, respectively), and both were linked to advanced stage and lymph node metastasis.

**Conclusion:**

The study concludes that PARD6G-AS1 hypomethylation and CD44 overexpression are potential predictors of recurrence in CN-H and CN-L EC patients, respectively.

## 1 Introduction

Endometrial cancer (EC) is one of the most common global malignancies of the female genital tract (Cho et al., 2019). Histologically, it mainly consists of endometrioid types exhibiting a favorable prognosis, whereas non-endometrioid types, such as serous and clear cells, have a high risk of recurrence (Siegel et al., 2020). However, the clinical outcome of EC cannot be discerned based on histological subtypes. Since the monumental publication of The Cancer Genome Atlas (TCGA) data in 2013, molecular classification has rapidly replaced the traditional histopathological classification of EC (Levine, 2013). According to TCGA, there are four molecular subtypes of EC: pathogenic somatic mutations in the exonuclease domain of the replicative DNA polymerase epsilon (POLE) ultra-mutated, microsatellite instability-hypermutated (MSI), copy number-low (CN-L), and copy number-high (CN-H). Distinct prognostic features of these subtypes have expanded their applicability in adjuvant treatment guidelines and various clinical trials (Van Den Heerik et al., 2020; Concin et al., 2021). Despite its improved prognostic and predictive performance, molecular classification is not ideal. For example, the CN-L subtype is known to be a substantially heterogeneous group, and prognosis is heavily affected by mutations in catenin beta 1 (CTNNB1) or overexpression of L1 cell adhesion molecule (L1CAM) (Kurnit et al., 2017; Kommoss et al., 2018). Therefore, it is necessary to identify potential biomarkers to complement the molecular classification. Furthermore, it would be of value to investigate another molecular signature to predict recurrence and better reflect tumor heterogeneity within each molecular subtype.

The advent of next-generation sequencing and high-resolution mass spectrometry technologies has facilitated large-scale multi-omics analyses, including genomic, epigenomic, transcriptomic, proteomic, and metabolomic research (Lu and Zhan, 2018; Olivier et al., 2019; Subbannayya et al., 2021). Omics technologies can be broadly applied in basic research and oncology clinical practice. Additionally, omics-based profiling can identify various molecular subtypes essential for personalized therapies. Recently, several studies have focused on distinguishing patients with cancer with different outcomes using multi-omics data (Chaudhary et al., 2018; Murugesan and Premkumar, 2021; Zhao et al., 2021). Ten long non-coding RNA models of bladder cancer have been identified as potential biomarkers based on multi-omics analysis (Xu et al., 2021).

Three mitochondrial genes, namely, HIGD1A, SUCLG2, and SLC25A24, have been associated with a poor prognosis of colorectal cancer via integrated analysis of the transcriptome and proteome (Zhang et al., 2021). Moreover, novel insights into tumor mutation burden-related gene expression signatures have been constructed using multi-omics data analysis in ovarian cancer. Both overall survival and disease-free survival-related prognostic models constructed based on tumor mutation burden-related genes show reliable predictive performance (Liu et al., 2020).

Furthermore, as part of artificial intelligence, machine learning improves the accuracy of cancer survival prediction models. Machine learning is a process that analyzes big data and can learn from mistakes and experiences (Schlick and Portillo-Ledesma, 2021). Several machine-learning models have been widely used to develop prediction models based on medical records, images, and molecular features of various malignancies (Ganggayah et al., 2019). Based on machine learning technologies, multi-omics data analysis offers a further understanding of predictive and prognostic phenotypes, facilitates the clustering of cancer samples into biologically significant groups, investigates the response to therapy, and serves translational research using integrative models (Chakraborty et al., 2018; Nicora et al., 2020; Subbannayya et al., 2021). Multi-omics data analysis using machine learning techniques for developing recurrence/survival prediction models has not been conducted.

This study retrieved three omics datasets from TCGA, including DNA methylation, RNA-sequencing, and variants. We merged them with machine learning analysis and investigated recurrence-related molecular signatures according to four molecular subtypes in EC.

## 2 Materials and methods

### 2.1 Study subjects and data source

The omics and clinical data of this study were downloaded from the TCGA UCEC (uterine corpus endometrial carcinoma) dataset. Downloads and data processing were performed using the R package TCGAbiolinks and the GDCquery function with the parameters listed in Table 1. All analyses were performed using the R package version 4.1.1. Information on the four subtypes was obtained from the supplementary data of a paper published by The Cancer Genome Atlas Research Network in Nature in 2013 (Levine, 2013). Omics and clinical data were organized in the R database format. Variant information is shown in *. maf format. We converted this into a matrix form comprising genomic location and patient ID.

**TABLE 1:** Parameters of GDCquery function from R package TCGA biolinks library.

Categories	Parameters	Data type	Workflow type
Project	TCGA-UCEC	TCGA-UCEC	TCGA-UCEC
Data category: DNA methylation	DNA Methylation	Methylation Beta Value	Liftover
Data category: RNA-seq	Transcriptome Profiling	Gene Expression Quantification	HTSeq - FPKM
Data category: variants	Simple Nucleotide Variation	Annotated Somatic Mutation	MuTect2 Annotation

### 2.2 Sample classification criteria

This study aimed to select the classification and stratification factors for each histological type (grade 1 endometrioid, grade 2 endometrioid, grade 3 endometrioid, and serous). We used three omics datasets: RNA sequencing, DNA methylation, and variants. RNA-sequencing data consisted of fragments per kilobase of transcript per million mapped reads (FPKM) values, and normalization was performed with a minimum value of 0 and a maximum value of 1. From the *.maf format, the converted matrix has values of 0 and 1, where one means that there are variants in the genomic location or gene, and 0 indicates no variants.

### 2.3 Bioinformatic analysis

For RNA-sequencing and DNA methylation analysis, two omics datasets were used as continuous variables, and the t-test was performed in two groups according to recurrence. Two omics data were read in R in matrix format, and fold change and p values were obtained for each gene by the “wilcox.test” R default function. The total fold change and p values were visualized as volcano plots, and heatmaps were plotted using the “pheatmap” R package.

Differentially expressed genes (DEGs) and differentially methylated regions (DMRs), and variants were used to select features for the machine-learning model. DMRs and DEGs were selected based on the fold-change thresholds and p values. The Fisher t-test, “fisher.test” in the R default function, selected the statistical significance of variants. Decision trees and random forest were the two machine-learning methods used to design the classification models in this study. Decision trees and random forests were applied using the “rpart” and “random forest packages”, respectively. The number of pre-trained tree models in the random forest analysis was 500. The top 30 genomic and clinical features were listed for the variable importance plot. The Gini importance was calculated as the average purity of the given genes. Enrichment analysis was performed using the “pathfindR” package, and enrichment terms were subsequently retrieved as an upset plot and heatmaps of enrichment terms. Survival analysis and survival curves were presented through R’s “survival” and “survminer” packages. The final follow-up date and recurrence status were set as events. The results analyzed with the “survfit” function were visualized with the “ggsurvplot” function. Statistical significance was estimated by the log-rank test between the two groups. All parameters used default values.

### 2.4 Validation using patient samples

To validate TCGA RNA-seq data, we performed RNA-seq using 16 tumor tissue samples from patients with endometrial cancer who underwent surgery at Korea University Guro Hospital (Approval number: KUIRB-2020–0191–01). From the tissues, total RNA was extracted using a TRIzol reagent. RNA samples were diluted in RNase-free water, and quality was assessed by gel electrophoresis. RNA samples with a RIN score greater than seven were used for RNA-Seq library construction. From isolated RNA, cDNA synthesis and NGS library preparation were performed using the Illumina SureSelect Library Preparation Kit version 2 (Illumina) by the manufacturer’s protocol. Paired-end sequencing was performed, and the read length was 101 bp. Aligned reads were produced by HISAT2 (version 2.2.0), and transcript assembly was performed by StringTie (version 2.2.0). The reference genome version was hg38 and calculated FPKM values were used for further analysis (GSE271198).

To validate TCGA methylation data, we analyzed Twist Methylome from 10 endometrium cancer tissues of Korea University Guro Hospital (GSE271199). All DNA samples were extracted using DNeasy Blood & Tissue Kits (QIAGEN). Then, DNA samples were sheared to a length of 200–250 bp (ME220 Focused-ultrasonicator, Covaris). Library construction was performed by NEBNext^®^ Enzymatic Methyl-seq Kit. The sequencing reads were produced by the Illumina platform. The adapter sequences are trimmed off the raw sequence reads and filtered by quality. The trimmed reads are mapped to the reference genome (hg38) with BSMAP (version 1.0). The only uniquely mapped reads are selected to sort and index. PCR duplicates were removed by SAMBAMBA (version 0.5.9). The methylation ratio of every single cytosine location within the on-target region was selected from the mapping results using the ‘methylatio.py’ script in BSMAP. The results of the coverage profiles were calculated as the “number of C/ effective CT counts” for each cytosine in CpG, CHH, and CHG. Each cytosine locus in CpG, CHH, and CHG is annotated using the table browser function of the UCSC genome browser. Annotation includes the functional location of each gene (promoter regions, which are defined as upstream 2 kb of the transcription start site, exons, and introns), transcripts ID, gene ID, strand, and CpG island.

## 3 Results

**FIGURE 1:**
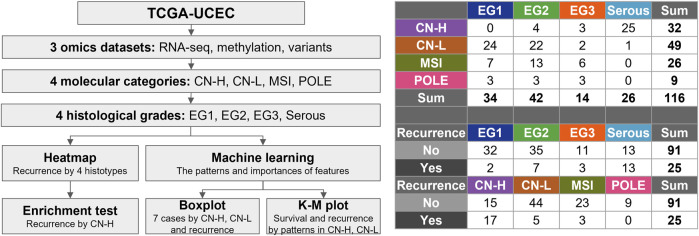
Flowchart for selection of the subjects. The 116 samples were classified into four molecular categories (copy-number high; CN-H, copy-number low; CN-L, microsatellite instability; MSI, polymerase epsilon; POLE) and four histological grades. Four histological grades consist of endometrioid grade (E.G.,) 1, 2, 3, and serous.

The process of this study is illustrated in Figure 1. Three omics datasets containing clinical data were obtained. 116 subjects were classified into four molecular categories and four histological grades. The landscape of the three omics datasets is depicted as a heat map (Supplementary Figure S1). The heatmap presents the molecular categories, histological grades, vital status, and recurrence as column annotation bars. In total, 568,845 genomic features were identified. 378,278 beta values defined the DNA methylation patterns. As a result of RNA-seq, 53,409 genes with FPKM values normalized to a value between 0 and one were identified. Variant information is presented as a value of 0 and one for each of the 118,555 genomic locations. 18,603 genes with at least one variant in one gene were presented as 0 and 1.

### 3.1 Select DMRs, DEGs, and variants from three omics data between recurrences

DMRs and DEGs between the recurrence and non-recurrence groups were selected by t-test. In all DMR analyses, CpG sites satisfying |fold change| > 0.18 and p < 0.0005 were selected (Supplementary Figure S2). Genes that were located at each CpG site were named and mapped; otherwise, they were indicated by an accession number (cgxxxxxxxx). Hypermethylated CpG sites in the recurrence group are indicated in red on the upper right of the volcano plot. Excluding the results of 32 samples belonging to CN-H, there were many hypermethylated CpG sites in the non-recurrence group under the three comparative conditions in the DNA methylation data (Supplementary Figures S2A–D). Higher expression patterns of DEGs were detected in the non-recurrence group in the RNA-seq data (Supplementary Figure S2E–H).

**FIGURE 2:**
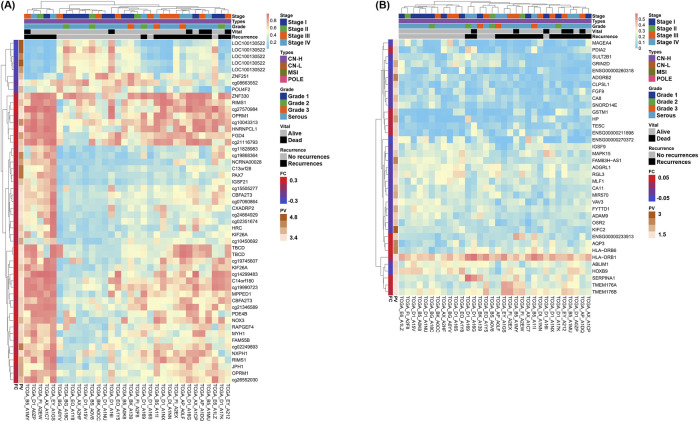
Heatmaps of differentially methylated regions (DMRs) and differentially expressed genes (DEGs) by recurrences of two omics datasets in copy-number high (CN-H; n = 32) groups. Genes satisfying the cut-off threshold, indicated in red or green in the Volcano plot, were presented as heatmaps. Five column annotation bars indicate stage, molecular subtypes, histological subtypes, vital status, and recurrence. In the two row annotation bars, the red line of fold change indicates hypermethylation or overexpression in the recurrence group. Statistical significance is presented as the p-value converted to log10, with higher values depicted with darker colors. **(A)** Heatmap of DMRs in CN-H group. 55 DMRs showed a p < 0.0005 and satisfied |fold change| > 0.18. Among DMRs, 46 and nine genes were hypermethylated or hypomethylated in accordance with recurrence, respectively. **(B)** Heatmap of DEGs in CN-H group. 37 DEGs showed a p < 0.05 and satisfied |fold change| > 0.05. Among DEGs, 12 and 25 genes showed higher expression or lower expression in accordance with recurrence, respectively.

Heatmaps were generated for the two groups to present the methylation or expression levels. Column annotation bars indicate four histological subtypes and recurrences. Row annotation bars indicate fold change and p value on a log10 scale. The closer the fold change in the row annotation bar to the red line, the more hypermethylated or overexpressed the gene in the recurrence group, and the darker the p-value, the more statistically significant the gene. Interestingly, both omics results confirmed that the recurrence and non-recurrence groups were well clustered only in the CN-H group among the four conditions in the heatmaps (Figure 2).

Heatmaps for 116 samples, including four histological classifications, were provided according to recurrence for DMR (Supplementary Figure S3A) and DEG (Supplementary Figure S3B). Two heatmaps were created, including the genes indicated in red or green in the volcano plot for DMR (Supplementary Figure S2A) and DEG (Supplementary Figure S2E), comparing 91 non-recurrences and 25 recurrences. A total of 44 and 48 genes were identified in the DMR and DEG, respectively. Clustering patterns were observed, but recurrence and non-recurrence could not be distinguished well in 116 samples.

In contrast, clustering according to recurrence was more successful in the DEG and DMR analyses of 32 CN-H patients. 46 DMR and 42 DEG were identified (Figure 2). According to the column annotation bar of each heatmap, patients included in the recurrence groups were classified as high-stage, serous, or endometrioid-type grade 3. Heatmaps were also provided for 49 and 26 patients with CN-L and MSI, respectively (Supplementary Figure S4). The number of patients with recurrent CN-L and MSI was five and three, respectively, and clustering was observed, but there was no remarkable pattern due to the low ratio of the total patients.

We obtained a list of results from the Kyoto Encyclopedia of Genes and Genomes (KEGG) pathway map (Supplementary Figure S5) and gene ontology (GO) analyses of recurrences in the CN-H groups (Supplementary Figure S6). The results of KEGG analysis indicated that these DMRs were enriched mainly in the neuroactive ligand-receptor interaction and PI3K-Akt signaling pathway. DEGs were enriched mainly in the cGMP-PKG signaling pathway, adrenergic signaling in cardiomyocytes, and cAMP signaling pathway. The cAMP signaling pathway was detected in both the DMRs and DEGs results.

### 3.2 Machine learning analysis of DEGs and DMRs between recurrences in CN-H and CN-L samples

**FIGURE 3:**
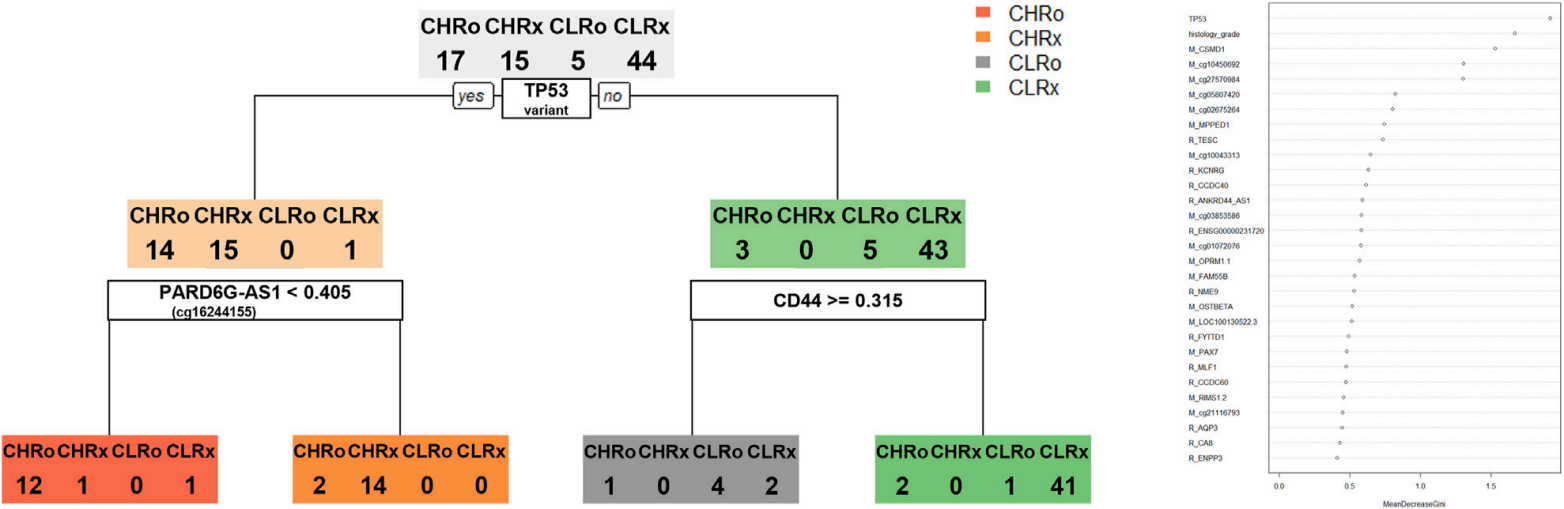
Decision tree and random forest model for classifying four groups of copy-number high and low according to the recurrence. Four groups consisting of copy-number high recurrences (CHRo), copy-number high non-recurrences (CHRx), copy-number low recurrences (CLRo), copy-number low non-recurrences (CLRx). **(A)** The decision tree model provides three nodes to classify four groups. **(B)** Top 30 features found by Gini importance were listed in the random forest model to classify four groups.

**FIGURE 4:**
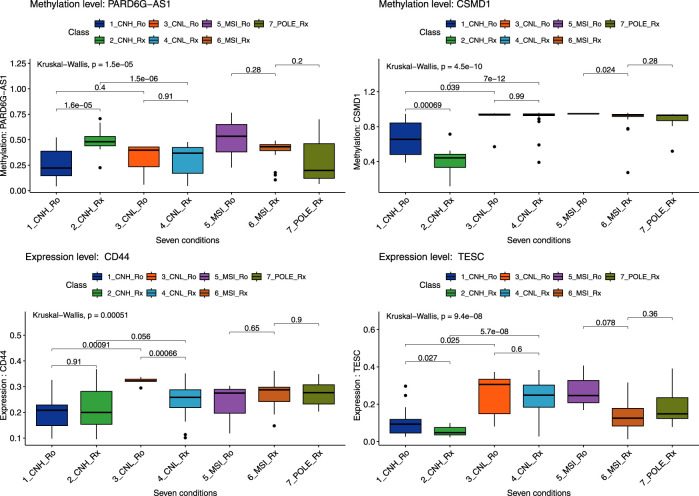
Boxplot of six genes by seven conditions by random forest model. A total of seven conditions indicates four pathological types with recurrences. Seven conditions consist of copy-number high recurrences (CNH_Ro), copy-number high non-recurrences (CNH_Rx), copy-number low recurrences (CNL_Ro), copy-number low non-recurrences (CNL_Rx), microsatellite-instability recurrences (MSI_Ro), microsatellite-instability non-recurrences (MSI_Rx), and POLE non-recurrences (POLE_Rx). Statistical significance between the two groups and for the entire group was secured through the Kruskal–Wallis test, and the p-value was presented.

We selected DEGs and DMRs between recurrences in the CN-H and CN-L analyses to design machine learning-based models. To design the model, DMRs and DEGs according to recurrence in each CN-H and CN-L group, and specific variants were selected. In the methylation analysis results, 95 DMRs (CN-H = 46 and CN-L = 49) were found, of which 87 were used for model construction. As a result of RNA-seq analysis, no common genes existed between each group, and 88 DEGs, 42 and 46, were used for model construction. According to the results of the variant analysis, 49 cases were significantly distributed according to the presence or absence of recurrence in CN-H (Fisher t-test result odds ratio <0.87 or >2), and 265 cases in CN-L (Fisher t-test result odds ratio <0.2 or >5), 310 belonging to the union were used in the analysis. 485 features were visualized as a heat map (Supplementary Figure S7). Decision-tree analysis revealed the four groups of CN-H and CN-L with recurrences (Figure 3). The CN-H and CN-L groups were classified according to TP53 variants. The methylation level of PARD6G-AS1 in the CN-H node and the expression of CD44 in the CN-L node are differentiated from recurrence (Figure 3A). Four groups of high and low copy numbers due to recurrence were classified using the random forest model, and the top 30 features found by Gini importance are listed (Figure 3B). To validate the three nodes of decision trees and top genes with the Gini index from random forest results, PARD6G-AS1 and CSMD1 methylation and CD44 and TESC expression under seven conditions were visualized as boxplots (Figure 4). The seven conditions consisted of CN-H recurrence and non-recurrence, CN-L recurrence and non-recurrence, MSI recurrence and non-recurrence, and POLE non-recurrence. As seen in Figure 4, methylation level of PARD6G-AS1 was significantly decreased in CN-H recurrence than that of CN-H non-recurrence, methylation level of CSMD1 was significantly increased in CN-H recurrence than that of CN-H non-recurrence, and the expression level of TESC was increased in CN-H recurrence than that of CN-H non-recurrence (P < 0.001, P < 0.001, and P = 0.027, respectively). In the CN-L group, the expression level of CD44 was significantly increased in patients with recurrence than those with non-recurrence (P < 0.001).

### 3.3 Validation of predictability of biomarkers in terms of recurrence using TCGA clinical data and institutional surgical specimens

**FIGURE 5:**
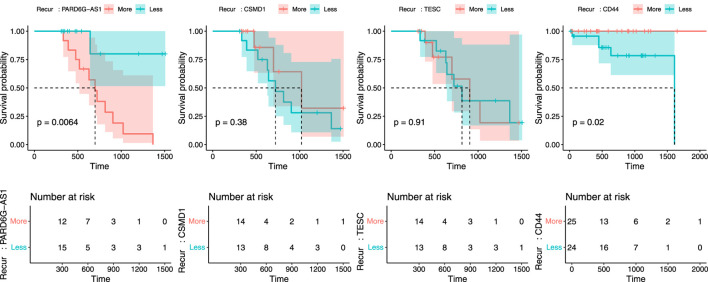
Recurrence plot to validate the predictability of PARD6G-AS1, CSMD1, TESC, and CD44 in relation to recurrence using TCGA clinical data. **(A)** PARD6G-AS1, **(B)** CSMD1, **(C)** TESC, **(D)** CD44.

Next, we tried to validate the results retrieved from machine learning analyses. To do this, a 2-step validation process was conducted. First, validation was performed using TCGA raw clinical data. From these data, we can analyze the actual usefulness of four biomarkers in predicting recurrence in CN-H and CN-L groups. As seen in Figure 5, among four biomarkers, only PARD6G-AS1 hypomethylation in CN-H and CD44 overexpression in CN-L showed statistical significance for disease recurrence (P = 0.006 and P = 0.02). The other two biomarkers (CSMD1 and TESC) failed to correlate with recurrence significantly. We also evaluated the association between two biomarkers and clinicopathological factors (Table 2). PARD6G-AS1 hypomethylation showed a statistically significant association with advanced stage and lymph node metastasis (P = 0.018 and 0.037, respectively). CD44 overexpression was also significantly associated with FIGO stage III/IV and lymph node metastasis (P = 0.014 and 0.013, respectively).

**TABLE 2:** Association between biomarkers and clinicopathological factors.

	PARD6G-AS1			CD44		
	Hypomethylation	Hypermethylation	p value	Low expression	High expression	p value
	(n = 49)	(n = 32)		(n = 72)	(n = 9)	
Age, median, year	63	67	0.487	64	58	0.532
BMI, kg/m^2^, n (%)			0.646			1
<30	22 (44.9)	12 (37.5)		30 (41.6)	4 (44.4)	
≥30	27 (55.1)	20 (62.5)		42 (58.4)	5 (55.6)	
Stage, n (%)			0.018			0.014
I-II	27 (55.1)	26 (81.2)		55 (76.3)	3 (33.3)	
III-IV	22 (44.9)	6 (18.8)		17 (23.7)	6 (66.7)	
Grade, n (%)			0.245			0.731
1–2	33 (67.3)	17 (53.1)		42 (58.3)	6 (66.7)	
3	16 (32.7)	15 (46.9)		30 (41.7)	3 (33.3)	
Histological type, n (%)			0.474			1
Endometrioid	34 (69.3)	19 (59.3)		47 (65.2)	6 (66.7)	
Serous	15 (30.7)	13 (40.7)		25 (34.8)	3 (33.3)	
Lymph node metastasis, n (%)			0.037			0.013
Negative	32 (65.3)	28 (87.5)		61 (84.7)	4 (44.4)	
Positive	17 (34.7)	4 (12.5)		11 (15.3)	5 (55.6)	

BMI; body mass index.

Finally, we performed a validation analysis using surgical specimens in our institution. In that experiment, the delta mean value of PARD6G-AS1 was −0.056, suggesting methylation level was decreased in patients with recurrence compared to those with non-recurrence in the CN-H group (P = 0.002). As for CD44, the absolute fold change value was 1.39, which meant the expression level was increased in patients with recurrence compared to those with non-recurrence in the CN-L group, but it did not show statistical significance.

## 4 Discussion

Integrative analysis and machine learning application to TCGA datasets (DNA methylation, RNA-seq, and variant dataset) revealed that PARD6G-AS1 hypomethylation was significantly associated with an increased risk of recurrence in CN-H EC. In addition, increased expression of CD44 was significantly associated with an elevated risk of recurrence in CN-L EC.

Among the four molecular subtypes of TCGA, the CN-H group has been regarded as a prognostically homogeneous entity owing to its poor prognosis (Santoro et al., 2021). The CN-H group consisted of various histological types, of which the serous type was the major component. In the PORTEC-3 trial, patients with high-risk features gained a survival benefit when they received adjuvant chemoradiation compared to those without radiation (León-Castillo et al., 2020). Moreover, this benefit is particularly evident in patients with serous EC. According to the 2021 European Society of Gynecological Oncology (ESGO)-European Society for Radiotherapy and Oncology (ESTRO)-European Society of Pathology (ESP) guidelines, the CN-H group with myometrial invasion was classified as a high-risk group requiring combined chemotherapy and radiation, irrespective of disease stage (Concin et al., 2021). National Comprehensive Cancer Network (NCCN) guidelines (version 1. 2022) also noted that adjuvant chemotherapy with or without radiation should be administered to patients with serous EC, except for a small population of serous histology with tumors limited to endometrium without myometrial invasion (https://www.nccn.org/guidelines/guidelines-detail?category=1&id=1473).

Furthermore, in the currently ongoing TransPORTEC refining adjuvant treatment in EC based on the molecular profile (RAINBO) umbrella trial (https://clinicaltrials.gov/ct2/show/NCT05255653), all CN-H participants received chemoradiation regardless of the FIGO stage. Collectively, receiving at least chemotherapy, preferentially with radiation, has been established as standard adjuvant management in patients with CN-H (or serous) EC. This is primarily due to the high risk of disease recurrence. However, not all CN-H EC recur. Nevertheless, the current adjuvant treatment strategy for CN-H EC is relatively uniform, so a tailored adjuvant treatment strategy might be needed based on the differential risk of recurrence within this group. In this regard, the methylation status of PARD6G-AS1 identified in this study could be a potential biomarker for deciding adjuvant treatment in CN-H EC. To the best of our knowledge, this is the first study to demonstrate methylation markers for predicting the recurrence of CN-H EC using integrative analysis and machine learning applications. PARD6G-AS1 was identified as a region containing DMR related to maternal imprinting. Perturbation of maternal imprinting in PARD6G-AS was associated with hematopoietic cancer (De Sá Machado Araújo et al., 2018). In addition, although there were differences in the CpG sites, the PARA gene has been detected as a factor related to early life stressors and aggressive behavior (van Dongen et al., 2015; Howard et al., 2022). PARD6G-AS1 was identified as LOC100130522 in the Illumina 450k methylation chip, and the DMR of PARD6G-AS1 in the cg16244155 probe was a factor for recurrence in CN-H groups. It has been noted that PAR6 proteins, including the PARD6G-AS1 gene, interact with classical cancer driver signaling pathways, such as MAPK and PI3K (Marques and Klefström, 2015). However, the effect of methylation on prognosis in cancers is not yet known. This study is meaningful for elucidating the prognostic effect of PARD6G-AS1 gene methylation in cancer for the first time. Further research is needed on the methylation of PARD6G-AS1 and related CpG sites in terms of mental health, cancer, and various chronic diseases.

CD44 is a cell surface glycoprotein involved in cell-to-cell interactions, adhesion, and migration (Spring et al., 1988; Leblanc et al., 2001). These characteristics are associated with cancer invasion and metastasis. In addition, CD44 overexpression has been associated with lymph space involvement and myometrial invasion in EC (Leblanc et al., 2001). Consistent with our results, a previous study found that CD44 was overexpressed in CN-L EC samples with recurrence and under-expressed in serous compared with endometrioid histology (Wojciechowski et al., 2015). CN-L EC is a heterogeneous entity and the largest group among the four TCGA molecular subtypes. Although this subtype characterizes low-grade endometrioid histology and harbors an intermediate prognosis, survival differs depending on the presence of specific biomarkers. For example, if L1CAM is overexpressed, its prognosis becomes detrimental, similar to CN-H (Kommoss et al., 2018).

Furthermore, CTNNB1 mutations identify low-grade patients with early-stage EC at an increased risk of recurrence (Kurnit et al., 2017). In addition to these two biomarkers, we present CD44 as a novel prognostic marker for predicting the recurrence of CN-L EC in this study. If CD44 is overexpressed, escalating adjuvant treatment can be considered for CN-L EC.

In this study, differential recurrences according to molecular subtypes were presented using an integrated omics dataset using machine learning techniques. Furthermore, we visualized genetic factors using machine learning approaches (e.g., decision trees), providing insight into cancer research. This could provide a methodology for integrated omics data analysis and clinical research into other cancers in the future. The genes and CpG sites presented in this study can also be used for research related to other diseases. This makes it possible to design disease models. To date, few studies have been performed that label recurrence in CN-H and CN-L groups using these machine-learning approaches, highlighting the uniqueness of our study. Another strength of our study is that we further validated the predictability of recurrence using both TCGA clinical data and our institutional surgical data.

PARD6G-AS1 hypomethylation and CD44 overexpression showed significant association with advanced FIGO stage and positive lymph node metastasis. These results suggest that unknown mechanisms of those two biomarkers related to negatively impacting the prognosis could exist. The verification experiment at our institution showed a similar pattern, but verification of CD44 expression level did not show sufficient statistical significance. Compared to DMR, the threshold is less stringent in DEG selection. Therefore, the selected genes have the possibility of being false positives. So, future work should focus on strengthening the model through large-scale, experimental verification. Additionally, for the first time, we present a methylation marker that can predict recurrence within the CN-H EC group, which has a relatively uniform adjuvant treatment.

A limitation of this study is its retrospective nature, resulting in surgical techniques, and the type of adjuvant treatment used could not be standardized. Therefore, we cannot exclude the possibility of these factors affecting recurrence. However, TCGA clinical data regarding the types of adjuvant treatment needs to be more extensive, limiting further analysis of the effects of adjuvant treatment on recurrence. Second, the study may be limited by the small sample size. 116 samples from all three omics datasets (methylation, RNA-seq, and variant) were retrieved and analyzed. This was approximately a quarter of the total number of patients in TCGA-UCEC (n = 548). Third, validation analysis using our institutional surgical samples failed to show a statistically significant association between CD44 overexpression and increased recurrence in CN-L EC, contrary to those results using TCGA clinical data. Because of the small sample number (n = 16), bias induced by individual differences might not have been overcome, leading to negative results compared to TCGA data. We assumed that all gene expression levels, or DNA methylation levels were normally distributed, but different distribution patterns were detected. Therefore, we need to further upgrade our code on the TCGA dataset and design a sophisticated model. To select DEGs and DMRs, we used the default R function, “t.test”. For T-test analysis, it must be assumed that the values of the two groups follow a normal distribution. However, gene expression and methylation levels are not normally distributed and usually show heterogeneity of variance among groups. So, there were some studies that confirmed the Wilcoxon-Mann-Whitney test to determine differences in expression and methylation levels (Michel et al., 2013; Hashim et al., 2014). In future research, a process of selecting DEGs and DMRs is needed through more sophisticated statistical techniques. Because there is a possibility of false positives and a high type I error rate for selected DEGs and DMRs, genes selected by a more stringent p-value could be provided.

Based on the three omics datasets of TCGA-UCEC, genetic and epigenetic factors that can distinguish recurrence were presented. Decision trees and random forests were used to classify and stratify the CN-H and CN-L samples by recurrence. Hypomethylation of PARD6G-AS1 in CN-H and CD44 overexpression in CN-L EC could predict disease recurrence. Based on our results, a differential adjuvant treatment strategy should be considered for CN-H and CN-L EC.

## Data Availability

The original contributions presented in the study are included in the article/Supplementary Material, further inquiries can be directed to the corresponding authors.
