# Supplementary material for: Integrative analysis of DNA methylation, RNA sequencing, and genomic variants in the cancer genome atlas (TCGA) to predict endometrial cancer recurrence
Source: Front Genet. 2025 Apr 28;16:1569122. doi: 10.3389/fgene.2025.1569122 (PMC12066751; doi:10.3389/fgene.2025.1569122)
Supplement: Supplementary file 1 [file Presentation1.pptx]

## Slide 1
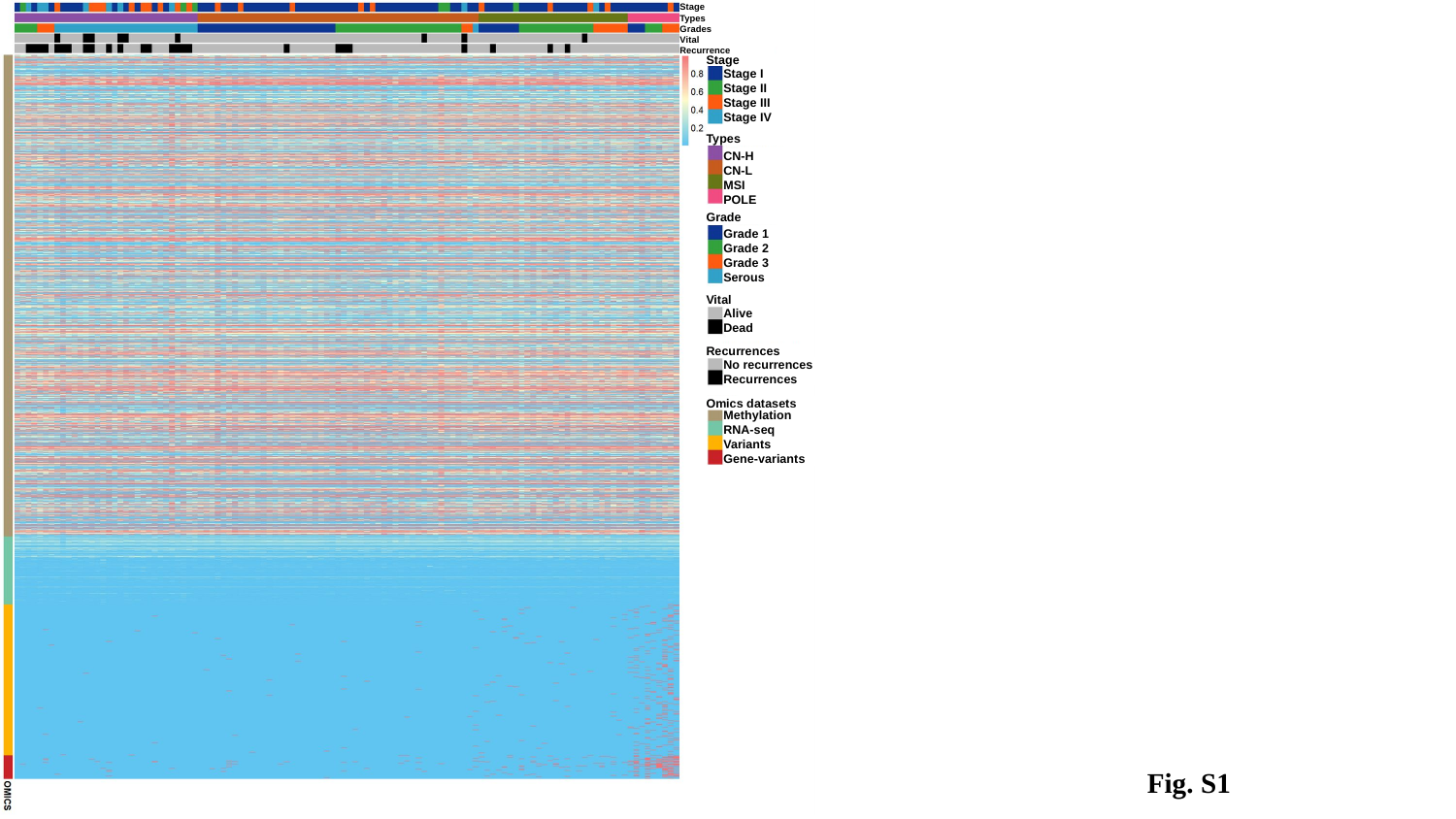

Stage
Types
Grades
Vital
Recurrence
Stage
Stage I
Stage II
Stage III
Stage IV
Types
CN-H
CN-L
MSI
POLE
Grade
Grade 1
Grade 2
Grade 3
Serous
Vital
Alive
Dead
Recurrences
No recurrences
Recurrences
Omics datasets
Methylation
RNA-seq
Variants
Gene-variants
Fig. S1

## Slide 2
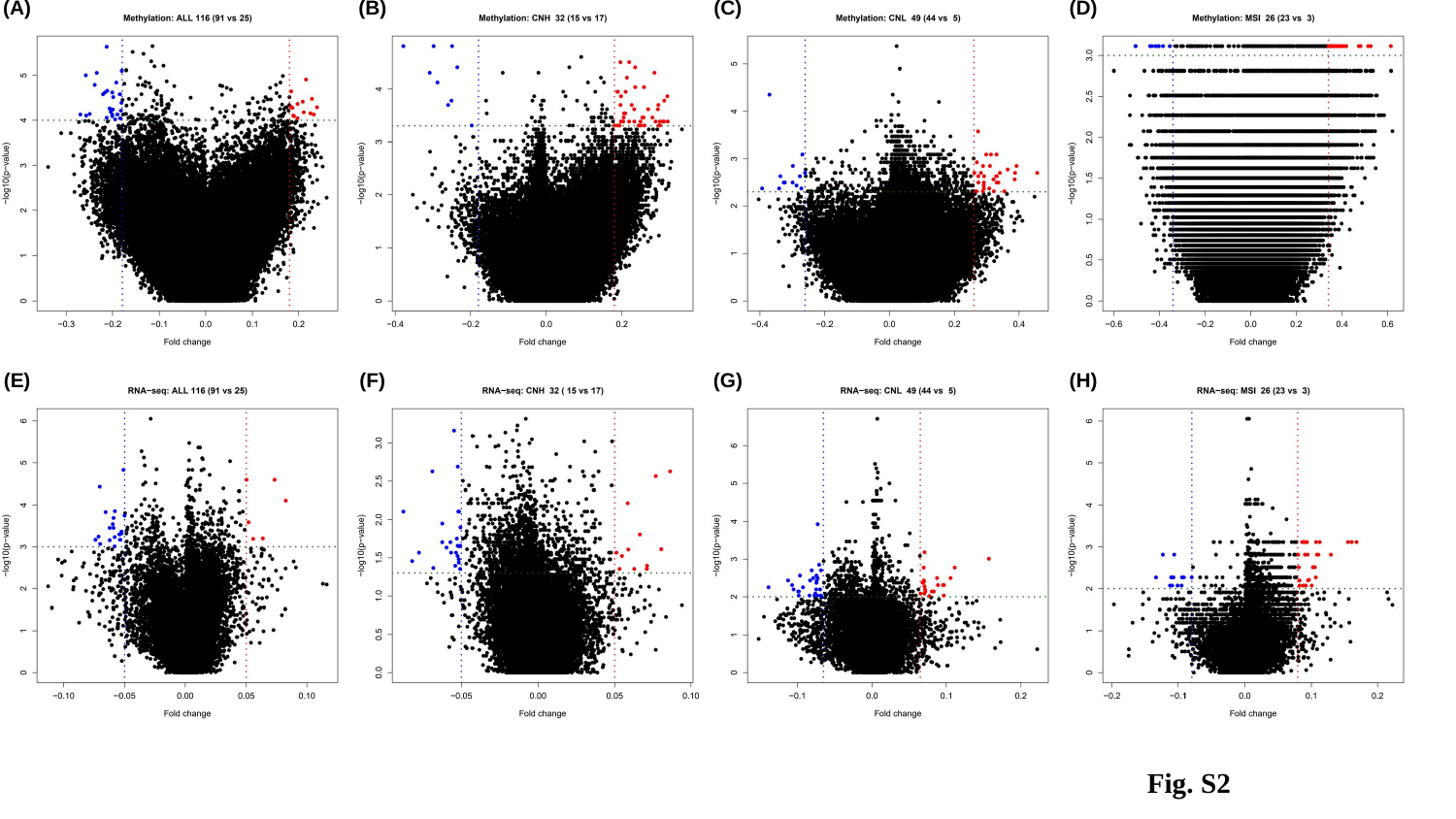

(A)
(B)
(C)
(D)
(E)
(F)
(G)
(H)
Fig. S2

## Slide 3
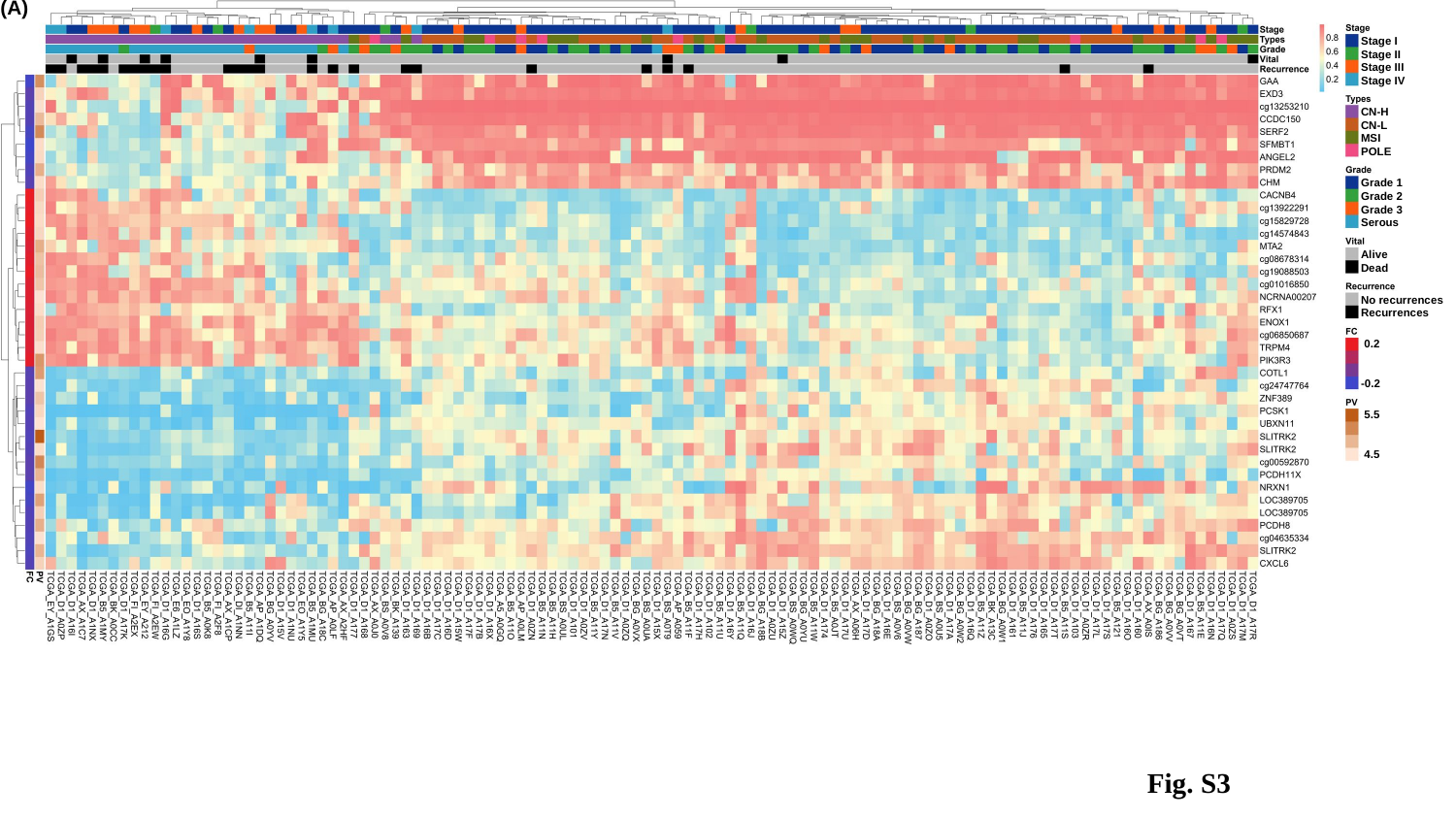

(A)
Stage I
Stage II
Stage III
Stage IV
CN-H
CN-L
MSI
POLE
Grade 1
Grade 2
Grade 3
Serous
Alive
Dead
No recurrences
Recurrences
 0.2
-0.2
 5.5
 4.5
Fig. S3

## Slide 4
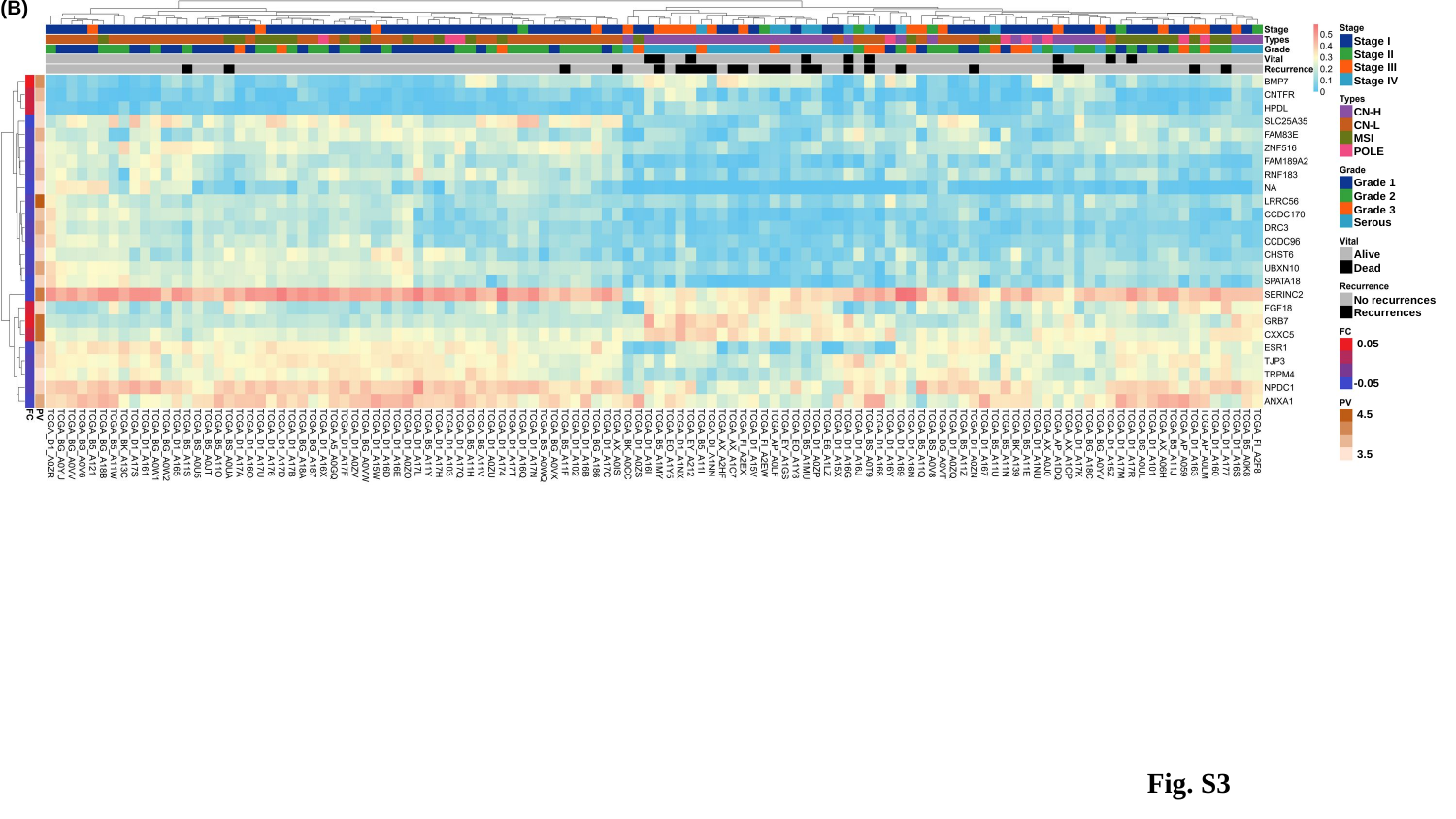

(B)
Stage I
Stage II
Stage III
Stage IV
CN-H
CN-L
MSI
POLE
Grade 1
Grade 2
Grade 3
Serous
Alive
Dead
No recurrences
Recurrences
 0.05
-0.05
 4.5
 3.5
Fig. S3

## Slide 5
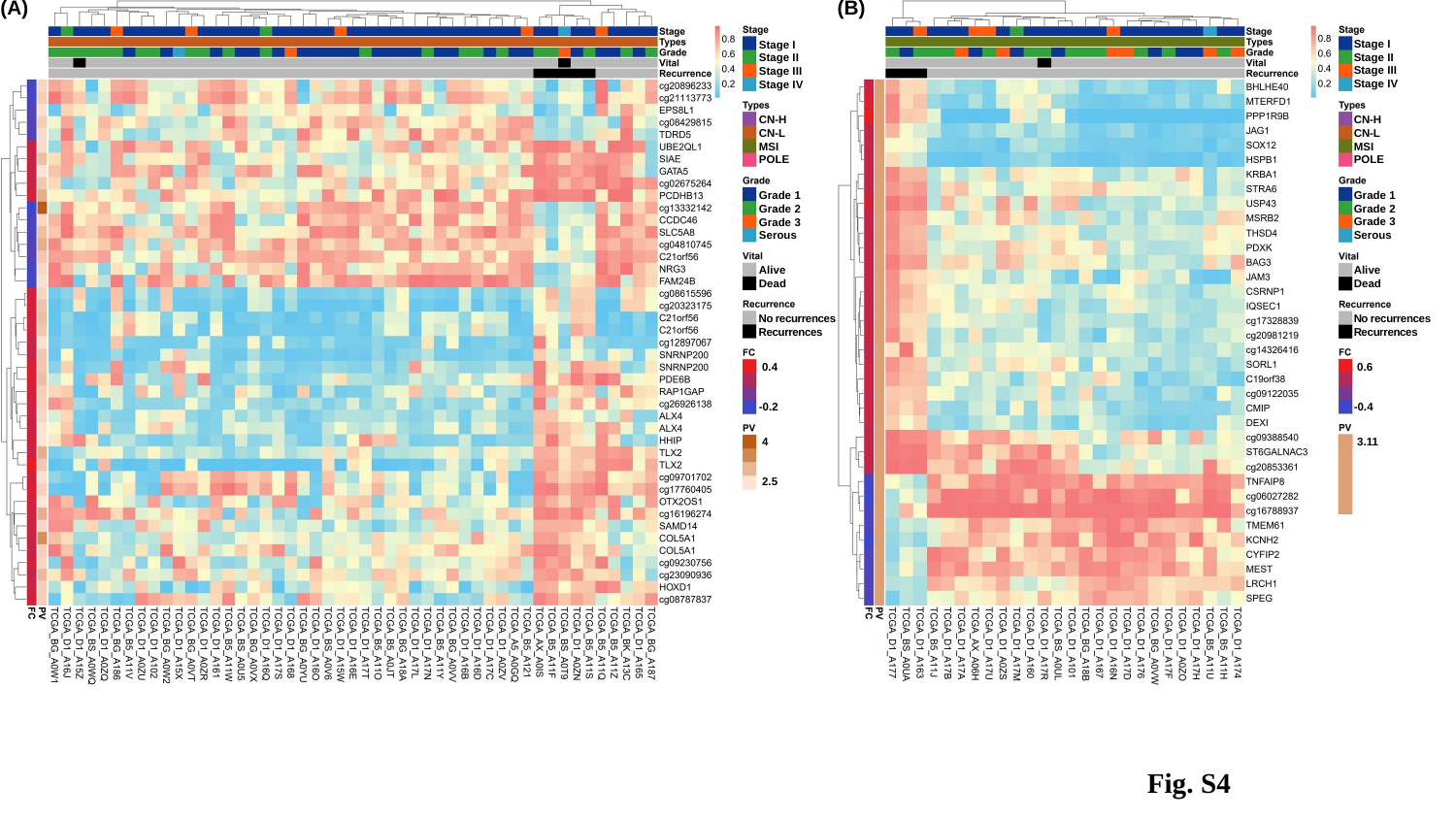

(A)
(B)
Stage I
Stage II
Stage III
Stage IV
Stage I
Stage II
Stage III
Stage IV
CN-H
CN-L
MSI
POLE
CN-H
CN-L
MSI
POLE
Grade 1
Grade 2
Grade 3
Serous
Grade 1
Grade 2
Grade 3
Serous
Alive
Dead
Alive
Dead
No recurrences
Recurrences
No recurrences
Recurrences
 0.4
-0.2
 0.6
-0.4
 4
 2.5
 3.11
Fig. S4

## Slide 6
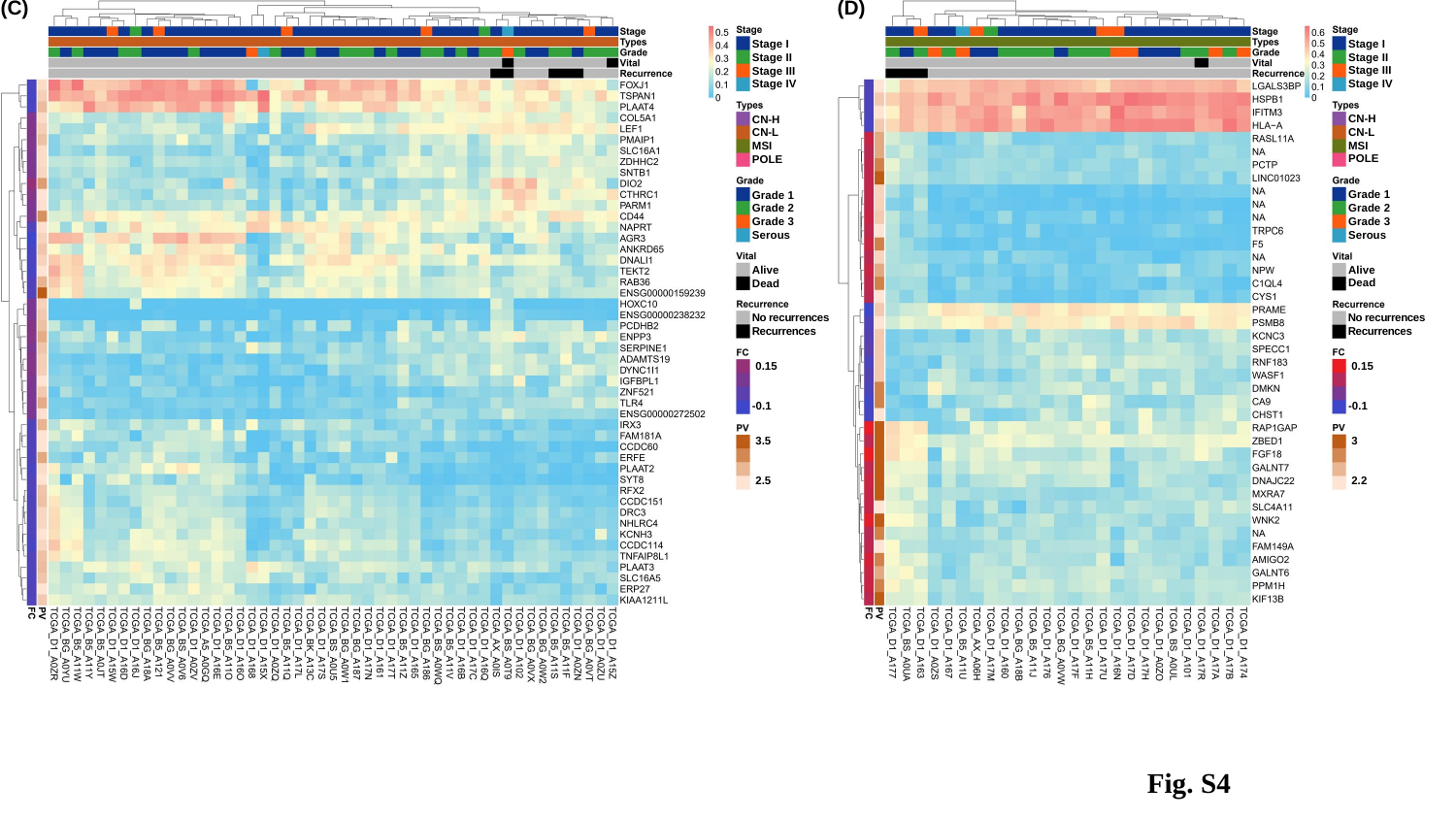

(C)
(D)
Stage I
Stage II
Stage III
Stage IV
Stage I
Stage II
Stage III
Stage IV
CN-H
CN-L
MSI
POLE
CN-H
CN-L
MSI
POLE
Grade 1
Grade 2
Grade 3
Serous
Grade 1
Grade 2
Grade 3
Serous
Alive
Dead
Alive
Dead
No recurrences
Recurrences
No recurrences
Recurrences
 0.15
-0.1
 0.15
-0.1
 3
 2.2
 3.5
 2.5
Fig. S4

## Slide 7
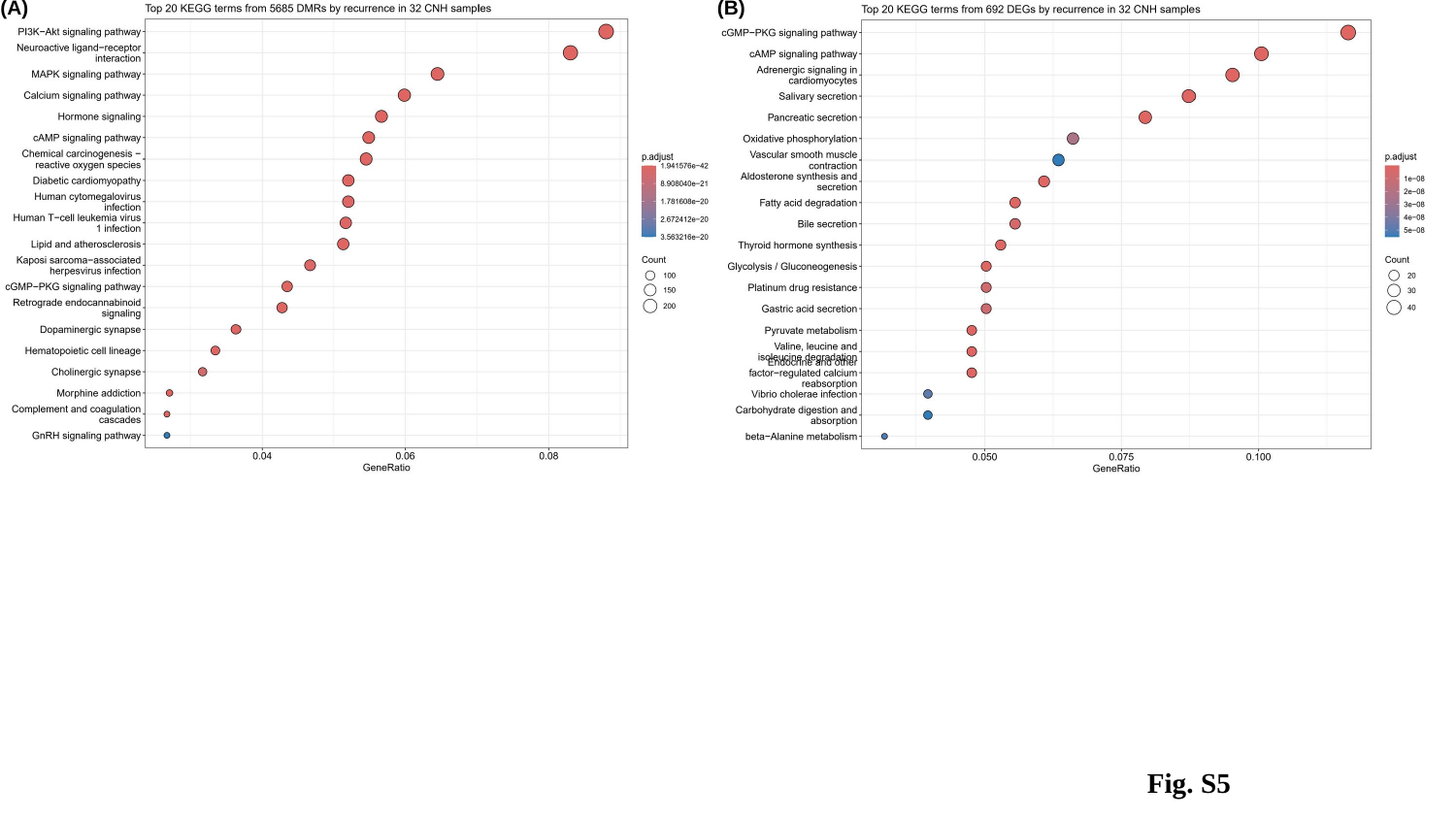

(A)
(B)
Fig. S5

## Slide 8
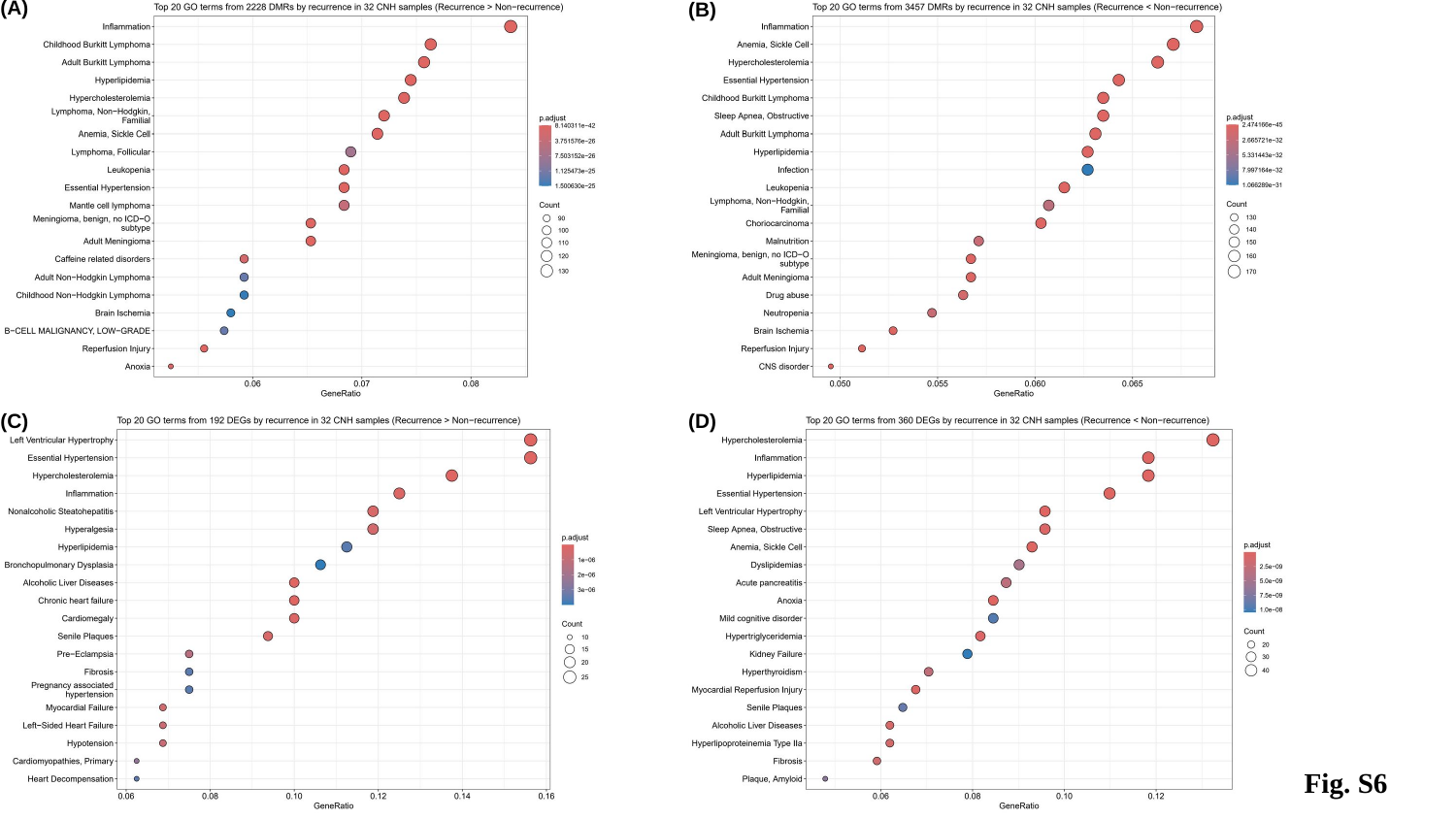

(A)
(B)
(C)
(D)
Fig. S6

## Slide 9
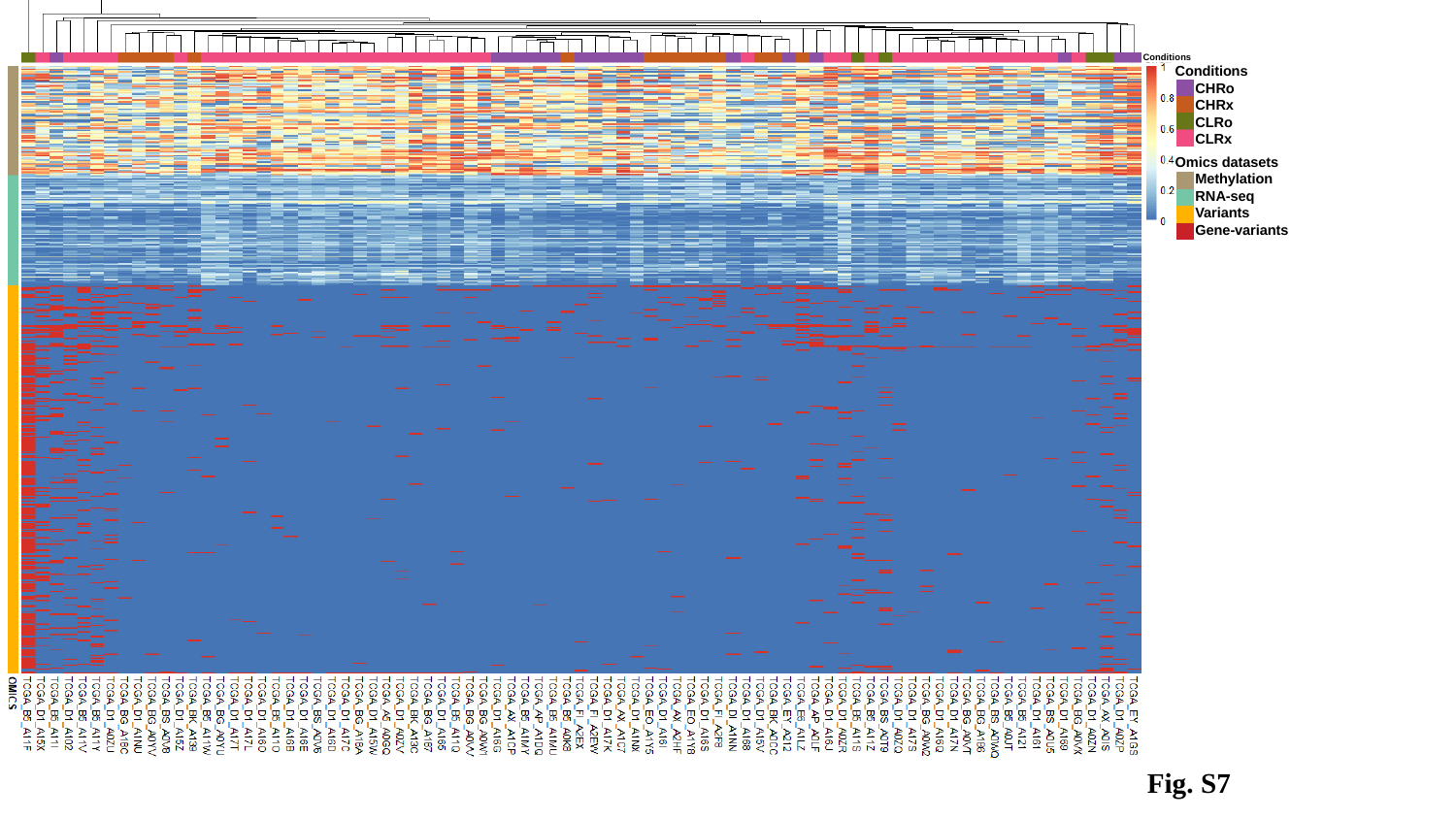

Conditions
Conditions
CHRo
CHRx
CLRo
CLRx
Omics datasets
Methylation
RNA-seq
Variants
Gene-variants
Fig. S7
